# Transcatheter Implantable Devices to Monitoring of Elevated Left Atrial Pressures in Patients with Chronic Heart Failure

**Published:** 2018-03-31

**Authors:** R. De Rosa, F. Piscione, D. Schranz, R. Citro, S. Iesu, G. Galasso

**Affiliations:** 1 Cardiology Department, “San Giovanni e Ruggi d’Aragona” University Hospital, Salerno, IT; 2 Hessen Pediatric Heart Center Giessen & Frankfurt, Goethe University Frankfurt, DE; 3 Cardiac surgery Department, “San Giovanni e Ruggi d’Aragona” University Hospital, Salerno, IT

**Keywords:** left atrium, heart failure, transcatheter devices

## Abstract

Elevated left atrial (LA) pressures are associated with poor prognosis in heart failure (HF). Invasive monitoring of LA-pressures and direct mechanical LA-decompression are associated with functional improvement in patients suffering from HF both with reduced and preserved ejection fraction. We aim to review the current available percutaneously implantable sensors for haemodynamic telemonitoring of LA-pressures (direct LAP sensor device-HeartPOD; right ventricular device-Chronicle; pulmonary artery device-CardioMEMs).

## Introduction

Heart failure (HF) is a leading cause of mortality and morbidity worldwide. Despite major advances in pharmacological and device therapy, hospitalisation rates due to cardiovascular causes in patients with HF remain high and did not show any significantly improvement over the last decades[1, 2]. Indeed, preventing acute decompensation in HF patients, both in those suffering from HF with reduced (HFrEF) and preserved (HFpEF) left ventricular ejection fraction (LV-EF) remains challenging. Numerous multivariable risk scores have been developed and may help to predict mortality, but have shown only a poor discriminative ability to predict subsequent cardiac hospitalization in patients with HF[3, 4]. Similarly, although a number of biomarkers related to cardiomyocyte stress in chronic HF have been identified in the last years[5–9], none of these have been showed to predict decompensation in routine clinical practice[10]. Haemodynamic studies have demonstrated that, irrespective of the underlying precipitant, the transition from chronic compensated to acute decompensated HF is characterized by distinguishing and progressive pathophysiological alterations[11, 12]. In particular, the increase in left atrial (LA) pressure represents the common pathway leading to pulmonary congestion and acute pulmonary oedema, that account for more than 90% of admissions because of acutely decompensated HF[13]. It has been shown that a progressive increase in LA pressure is apparent weeks before decompensation leading to hospital admission. On the contrary, classical symptoms and signs like dyspnoea, oedema or weight gain may occur only when the decompensation is incipient and mostly not avoidable anymore[11]. These pathophysiological observations lead to the hypothesis that a continuous haemodynamic monitoring of LA pressures may be useful to guide therapy of chronic HF and prevent acute decompensation. Along the same line, recent evidences[14–18] have shown that the “decompression” of LA through percutaneous implant of unidirectional left-to-right interatrial shunt devices is able to ameliorate symptoms in patients suffering from HF both with preserved and impaired LV-EF.

We aimed to review the knowledge regarding currently available implantable devices for direct and indirect haemodynamic telemonitoring of LA pressures in patients with chronic HF.

## Implantable device to monitor LA pressures

LA pressures (LAP) represents the best hemodynamic parameter to estimate the left ventricular filling pressures. However, in absence of a relevant mitral valve stenosis as well as in absence of precapillary pulmonary arterial hypertension, the pulmonary capillary wedge pressure (PCWP) closely correlates with end-diastolic arterial pulmonary pressure and can be used in spite of the hardly measurable LAP. Similarly, in absence of relevant pulmonary valve dysfunction, the right ventricular (RV) pressures closely remark the pulmonary artery pressures (PAP)[19]. Accordingly, three different devices were developed to estimation of left ventricular filling pressures: a direct LA pressure sensor, a right ventricular pressure device and a pulmonary artery pressure device.

## Direct left atrial pressure sensor device: HeartPOD device

The HeartPOD device (HeartPOD, Savacor, Inc, from St Jude Medical, Inc, Minneapolis, Minn, USA) was developed to be implanted in the left atrium and direct measure LA pressure, potentially providing more accurate assessment of left ventricular filling pressures, without potential confounding factors deriving from pulmonary vascular abnormalities.

### System and measurement

The HeartPOD left atrial sensing device consists of an implantable sensor coupled with a subcutaneous antenna coil and a patient advisory module (PAM). The sensor system is implanted into the atrial septum oriented to the LA and is fixed in the interatrial septum through two nitinol anchors; it included a 3×7mm sensor module hermetically sealed with a titanium pressure sensing membrane and circuity to measure LAP, temperature and intracardiac electrogram. LAP is obtained by subtracting the absolute pressure measured by the sensor from the atmospheric one measured by a pressure sensor located in the PAM. Measurement and calibration are performed through the skin by wireless transmissions from the PAM. The acquired waveforms are simultaneously stored in the PAM-memory.

### Delivery and implantation

The device is implanted through a right heart catheterization (femoral or subclavian venous accesses have been described until now). Transseptal atrial puncture is performed and an 11-F sheath is placed in the left atrium. The sensor system is advanced under fluoroscopic guidance until the distal anchor unfolds and contacts the left side of the interatrial septum; then, the sheath is withdrawn to release the sensor module. The antenna coil is connected to the proximal end of the sensor lead and placed in a subcutaneous pocket specifically created (usually, in the lower right rectus abdominus sheath in case of femoral access or in the pectoral sheath in case of subclavian access). After implantation, in patients not taking oral anticoagulants, dual antiplatelet therapy with acetylsalicylic acid (ASA) and Clopidogrel is recommended for a minimum of six months, followed by ASA-monotherapy lifetime. Measurement acquisitions are made by placing the PAM in proximity (< 5 cm) of the subcutaneous pocket.

### Current evidences

The safety and feasibility of the HeartPOD device in human settings were evaluated in the HOMEOSTASIS (Hemodynamically Guided Home Self-Therapy in Severe Heart Failure Patients) trial, a prospective, multicentre, observational open-label registry enrolling patients with chronic, symptomatic HF (HFrEF or HfpEF) and a history of acute decompensation. In the pilot HOMEOSTASIS study[20], the device was implanted in an initial population of 8 patients. After implantation, left atrial pressure, temperature and intra-atrial electrogram were registered twice daily for 3 months; a clinical assessment and a non-invasive device calibration were performed at 2, 6 and 12 weeks after implantation. After 3 months, patients underwent a new right heart catheterization, showing a good device accuracy, with 87% of device LAP measurements being within a 5 mmHg-deviation from simultaneously invasive measurements of pulmonary capillary wedge pressures. No device-related complications were observed. The safety and the accuracy of this novel interventional strategy were confirmed in the long-term report of the HOMEOSTASIS study[21], including 84 patients undergoing HeartPOD device implantation. No intraprocedural complications were observed, nor were there any specifically adjudicated to be device related during follow-up. The device showed a stable device-performance over a 4-years follow-up (median time: 14 months), with a considerable role of the learning curve in reducing device failure and measurement artefacts (from 12% in the first 43 patients to 0% in the last 41 patients enrolled), in particular through iterations in sensor design and implantation. Whether monitoring of LAP through this direct atrial sensor might translate in amelioration of symptoms and prognosis of patients with chronic HF still need to be investigated.

## Devices for indirect estimation of LA pressures

### Right ventricular pressure sensor-Chronicle device

The first implantable devices aiming to provide an invasive hemodynamic monitoring of patients with chronic HF were developed in the 1990 and estimated left ventricular filling pressures by hemodynamic parameters measured in the right ventricle.

#### System, implantation and measurement

**Figure 1:**
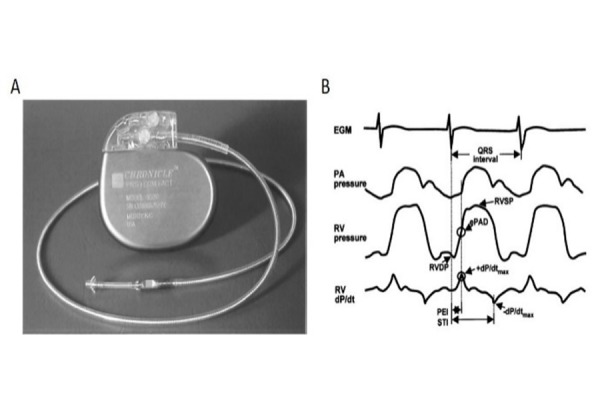
Right ventricular device (Chronicle) A: Right ventricular implantable device (Chronicle) with the pressure sensor at the distal tip of the single right ventricular lead; B: Representative waveforms obtained from the device: electrocardiogram, right ventricular (RV) pressure, pulmonary artery (PA) pressure and right ventricular dP/dt, ePAD: estimated pulmonary artery diastolic pressure; +dP/dt_max_: maximum positive dP/dt; -P/dt_max_: maximum negative dP/dt; PEI:pre-ejection interval; STI: systolic time interval. Reproduced with permission of [22]

The right-ventricular pressure sensor mostly used in the clinical research is the Chronicle (Chronicle, Medtronic, Minneapolis, Minn, USA) device. It is composed by a transvenous lead with a pressure-sensor at the tip and a programmable device to process and store information. It is implanted similar to a single-lead pacemaker, with the lead positioned transvenously in the right ventricular outflow tract or septum and the device positioned in a subcutaneous pocket created in the pectoral area. The Chronicle system continuously monitors and stores several information including heart fate, body temperature, patient activity, right ventricular systolic and diastolic pressures, change in right ventricular pressure, right ventricular pre-ejection and systolic time and estimated pulmonary arterial diastolic pressure (ePAD). This latter has been showed to be strongly correlated with actual pulmonary artery pressures, independently from loading conditions. Chronicle device and representative measurement images are shown in Figure 1.

#### Scientific evidence

A pilot study[22] including 32 patients with chronic HF in NYHA functional class II and III undergoing Chronicle device implantation showed that the systolic right ventricular pressure considerably increased 4 ± 2 days before occurrence of cardiac decompensation leading to hospitalisation. Grounded on such physiopathological bases, the randomized clinical trial COMPASS-HF (Chronicle Offers Management to Patients with Advanced Signs and Symptoms of Heart Failure)[23] was conducted on 277 patients with chronic HF. Patients eligible to enrolment should be in NYHA functional class III or IV despite ≥ 3 months of optimal medical therapy and have experienced at least one episode of cardiac decompensation in the previous 6 months. Both HFrEF and HFpEF clinical conditions were included. All the study patients underwent Chronicle-implantation and were thereafter randomized to treatment group (weekly acquisitions of the hemodynamic parameters and appropriate therapy-management) or control group (no acquisitions of the hemodynamic parameters). Implantation was successful in 274 (98.9%) patients. In successfully implanted patients, 23 (8.3%) procedure-or device-related complications occurred, leading to prolonged hospitalisation. However, all complications were successfully resolved and resulted in a fully functional month follow-up, a not significant reduction in HF-related events (including hospitalizations and emergency or urgent care visits requiring intravenous therapy) was observed in the treatment group (21% decrease in event rate versus control group, p=0.33). A secondary analysis investigating the time to the first hospitalisation because of acutely decompensated HF after randomization conversely showed a significantly longer event-free survival in patients in the treatment group compared with controls (36% reduction in the relative risk of HF-hospitalisation, p=0.03). Since the event rate in the control group was lower as expected (0.85 per 6 patient-months instead that 1.2 per 6 patients-months), making the study under-powered to detect significant differences in clinical events rate, studies on larger population are needed to assess whether a therapeutic regimen guided by haemodynamic monitoring with right ventricular implantable device may be helpful in reducing acute decompensation in patients with chronic HF.

### Pulmonary artery pressure sensor-CardioMEMS device

#### System and measurement

The CardioMEMS Heart Failure Sensor (CardioMEMS Inc., St. Jude Medical, Atlanta, Georgia) consists of three components: the implantable HF sensor, the delivery catheter and an external electronic monitoring unit connected with an external antenna. The implantable HF sensor is a miniature electrical circuit composed from a three-dimensional coil and a pressure-sensitive capacitor hermetically sealed and enclosed in a fused silica capsule. The device is 15-mm long and 3-mm wide and contains two nickel-titanium wire loops attached to each end that ensure a stable sensor position in the pulmonary artery. Pressure changes in the implantable sensor environment result in correlated changes to the circuit’s resonant frequency. The coil allows for electromagnetic coupling to the sensor by an external antenna, which is held against the patient’s chest approximately in the area of sensor deployment. The antenna provides power to the device and allows for continuous measurement of its resonant frequency, which is converted in a real-time pressure waveform on the electronic monitoring unit. The electronic unit is used by the physician to calibrate the HF sensor during implant and, after implantation, to take readings of hemodynamic data. The haemodynamic data provided are the following: PA pressure waveform; systolic, diastolic and mean PA pressure; heart rate. These data are transmitted to a secure Internet database where they are stored and made available for later reading.

#### Delivery and implantation

**Figure 2:**
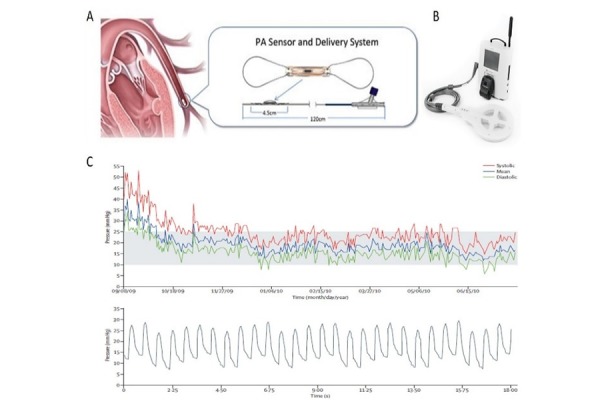
Pulmonary pressure sensor device (CardioMEMS) A: CardioMEMS sensor and delivery catheter and schematic representation of the implanted sensor; B: external electronic monitoring unit connected with antenna used to calibrate the device and to take readings of hemodynamic data; C: representative haemodynamic data provided by the device, including pulmonary rtery pressure waveforms (systolic, mean and diastolic) and a pressure trend graph. Reproduced with permission of [24].

The implantable HF sensor is connected to a tether wire within a hydrophilic delivery catheter and is implanted during a right heart catheterization. Femoral venous access is routinely used, although a jugular access may be preferred in case of severe obesity. After venous cannulation with an 11-French sheath, a Swan-Ganz catheter is advanced into the left or right pulmonary artery tree under fluoroscopic guidance and a selective pulmonary angiography is performed in order to identify an appropriate target artery. Thereafter, a 0.018-inch guidewire is placed in the target vessel, the Swan-Ganz catheter is retired, and a 12-French sheath is advanced over the guidewire, allowing the introduction of the CardioMEMS delivery catheter. Once the target site is reached, the HF sensor, which is connected to the end of the delivery catheter, is released into the target artery. A selective angiography is indicated to test the correct position of the device and the presence of distal blood flow. The delivery system is removed and exchanged again with the Swan-Ganz catheter; pulmonary artery pressures are acquired through the Swan-Ganz catheter and used to calibrate the sensor. Full anticoagulation is recommended during the procedure to prevent catheters and devices’ thrombosis. Patients not taking oral anticoagulants are recommended to undergo dual platelet therapy with ASA and Clopidogrel for a month after procedure, followed by ASA-monotherapy lifelong. Patients on oral anticoagulant therapy are recommended to continue their treatment. Device components and representative acquired waveforms are shown in Figure 2.

#### Scientific evidence and current recommendations

The CHAMPION (CardioMEMS Heart Sensor Allows Monitoring of Pressure to Improve Outcomes in NYHA functional Class III Heart Failure Patients) study[24] was a prospective, multicentre, single-blind randomized clinical trial aiming to investigate the impact on of a PA monitoring-guided HF therapeutic management on outcome of patients with chronic HF. 550 patients with both HFrEF and HFpEF, who were in NYHA functional class III despite optimal medical therapy and who had at least one hospitalisation for decompensated HF within the previous 12 months were enrolled. CardioMEMS system for monitoring of pulmonary pressures was implanted in the entire study population; patients were thereafter randomized to a PA-monitoring guided HF therapy or to a control group, in which daily uploaded pulmonary pressures were not made available. The control group received all standard pharmacological and device-based available therapies recommended by the currently guidelines. After a mean follow-up of 15±7 months, patients randomized to PA monitoring-guided therapy showed a 37% reduction in HF-related hospitalisation compared with the control group (p<0.0001). Such results were confirmed in both subgroups of patients with HFrEF[25] and HFpEF[26]. The recently published long-term report of the CHAMPION trial[27] investigated the impact on outcome of “cross-over” of patients formerly in the control group to the PA-monitoring-guided group, confirming a significant decrease of rates of hospitalisation for HF (48%, p<0.0001) when this therapeutic strategy was applied compared with standard regimen. The CHAMPION trial also demonstrated a good safety profile of CardioMEMS, with 1 out 550 (0.2%) cases experiencing a failure of delivery-system (that required the remotion of the system through a snare), no episodes of pulmonary infarction or embolism and no events required removal of the sensor over the entire follow-up time. According with results of the CHAMPION trial, current ESC guidelines give a class IIb indication, with level of evidence B, for monitoring of PA pressures with CardioMEMS in patients with chronic HF who had been previously admitted because of decompensation and who remain symptomatic despite optimal medical therapy, in order to reduce the risk of recurrent HF hospitalisation[28].

## Conclusions

Continuous monitoring of LAP through implantable devices placed in left atrium, in right ventricle or in the pulmonary artery represents a promising strategy to dynamically optimize pharmacological therapy and prevent acute decompensation in these patients. Currently, only pulmonary artery pressure sensor (CardioMEMS) has been found to improve symptoms and significantly decrease rate of cardiac hospitalization and its use may be considered in clinical practice to prevent decompensation in symptomatic HF patients. On the same pathophysiological basis, the interventional creation of a restrictive atrial communication to improve symptoms and ameliorate loading condition in patients with chronic HF is currently under investigation.
